# A Comparative Review of Pakistan’s Leading Biomedical Academic Journals and the Key Factors behind their Success

**DOI:** 10.12669/pjms.41.3.11923

**Published:** 2025-03

**Authors:** Afifa Ehsan, Shaukat Ali Jawaid

**Affiliations:** 1 Prof. Dr. Afifa Ehsan Professor, Department of Oral Biology, Faryal Dental College, Lahore, Pakistan; 2 Shaukat Ali Jawaid Chief Editor, Pakistan Journal of Medical Sciences, Karachi, Pakistan

Academic journals are graded to aid institutions, researchers, publishers, and policymakers appraise their implications and scientific impact within the global research landscape. Analyzing the three leading biomedical academic journals of Pakistan that are indexed in Clarivate and enjoy impact factors is a challenging task; the Pakistan Journal of Medical Sciences (PJMS), Journal of the College of Physicians and Surgeons Pakistan (JCPSP), and Journal of the Pakistan Medical Association (JPMA).^1^ This review encompasses a critical examination of their scope, editorial standards, and impact in the field of medical journalism. Each of these journals plays a fundamental role in spreading healthcare research both locally as well as internationally. Nevertheless, they do so with different editorial practices, target audiences, and themes of focus. This audit presents an outline of the similarities and differences between PJMS, JCPSP, and JPMA. By reviewing their editorial methods, publication regularities, research worth, and impact factors, we can delve into how every journal affects medical research. This evaluation emphasizes the fortes and zones for upgradation, tendering valued viewpoints for researchers and policy-makers.

To begin with, PJMS is recognized for its comprehensive coverage of medical disciplines, concentrating on quality research which has resulted in its high impact factor. JCPSP in contrast, supports postgraduate education and training, publishing research that relates to clinical practice while JPMA stands out for its wide-ranging approach to public health issues, medical ethics, and policy deliberations, targeting translating research into clinical practice. When comparing infrastructural resources, all the three journals have assigned offices; nevertheless, the allocated office area is abundant for JCPSP, adequate for PJMS, and deficient for JPMA. In terms of human resources, JCPSP is well-staffed with sufficient resources, while PJMS and JPMA have adequate staffing. Financial and IT resources are appropriate across all the three journals. Each journal also offers specified workstations and staff available. Financial sovereignty differs, with JCPSP depending on CPSP, PJMS having self-sufficiency, and JPMA depending on PMA. Archiving systems are also present in all the three.

Similarities among JCPSP, PJMS, and JPMA are highlighted in Table-I.. All have OJS with JCPSP presenting a modified version. They all charge processing and publication fees and follow ICMJE guidelines, with their origin dating back to 1991 for JCPSP, 1984 for PJMS, and 1953 for JPMA. All are acknowledged by HEC/PMDC, indexed in databases such as PubMed, PMC, and Scopus, claim an assorted editorial board, and sustain a monthly publication frequency.^2^-^3^

**Table-I:** Key Similarities between JCPSP, PJMS, and JPMA

	Features	JCPSP	PJMS	JPMA
1.	Online Journal System	Present & Customized	Present	Present
2.	Processing Fee	Yes	Yes	Yes
3.	Publication Fee	Yes	Yes	Yes
4.	Impact Factor Journal	Yes	Yes	Yes
5.	Authorship Guidelines	ICMJE	ICMJE	ICMJE
6.	Origin Year	1991	1984	1953
7.	HEC/PMDC Recognized	Yes	Yes	Yes
8.	Included in PubMed/PMC/Scopus	Yes	Yes	Yes
9.	Diversity of the Editorial Board	Yes	Yes	Yes
10.	Frequency of Publications	Monthly	Monthly	Monthly

The differences between JCPSP, PJMS, and JPMA are illustrated in Table-II. JCPSP stands out by performing statistical review ahead of peer review unlike PJMS and JPMA, where statistical reviews are carried out where indicated. PJMS and JPMA do not demand raw data sheets. Asking authors for raw data and statistical analysis sheets is a rare practice, as it does not ensure preventing fraudulent practices. Unfortunately, those who commit fraud are usually ahead of these fraud prevention measures.

**Table-II:** Key Variances between JCPSP, PJMS, and JPMA

	Features	JCPSP	PJMS	JPMA
1	Pre-Submission Review	No	Yes	No
2	Statistical Review	Before peer review	After peer review if advised	After peer review if advised
3	Processing Fee Submission	After initial review	At the time of submission	At the time of submission
4	Fee Refund Policy	Non-refundable processing/publication fee	Non-refundable processing/publication fee	Non-refundable processing/publication fee
5	Processing Fee	Rs. 5000/	Rs. 5000/	Rs. 3500/
6	Output Data Sheets	Required	Not required	Not required
7	Registration With COPE	Not registered	Registered	Registered
8	Copyrights	With Journal	With Author	With Author
9	Publication of Case Reports	Published separately	Published in regular issue	Published in regular issue
10	Evidence-Based Report	Yes	Yes	Yes
11	Clinical Practice Article	Yes	No	No
12	DOAJ	Not registered	Registered	Registered
13	Ownership	CPSP	Individual	PMA
14	Model Being Followed	Institutional	Business model	Organizational
15	Record Keeping	Extensive	Per need	Extensive
16	Authors’ Names in IRB Letter	Required	Required if more than four	Required if more than six
17	Number of Authors	Not more than six for a single-center study	Not more than four	Not more than six for a single-center study
18	Acronym on Title Page	Present	Not present	Not present
19	Printed Copies	550 copies	Online only	Less than 50
20	Hard Copy provision to the Authors	Yes	No	Yes, on payment
21	Plagiarism Check Software	Turnitin	Ithenticate	Turnitin
22	Submissions Per Month	150	450	100
23	Rejection Rate	50%	80%	42%
24	Impact Factor (2024)	0.7	1.2	0.8
25	Student Section	No	No	Yes
26	Student Trainees	No	No	Yes
27	Review Process	Double-blind	Open	Double-blind
28	Honorarium To Reviewers	Yes	Discounts in publications	No
29	KAP Studies	Discouraged	Discouraged	Entertained

**Table-III:** Impact Factor of these three journals as per JCR-2024.

Name of the Journal	Impact Factor	Quartile Ranking
Pakistan Journal of Medical Sciences	1.02	Q2
Journal of Pakistan Medical Association	0.8	Q3
Journal of College of Physicians & Surgeons Pakistan	0.7	Q3

JCPSP charges processing fees after preliminary review, while PJMS and JPMA do so upon submission. JCPSP is exclusive in holding the copyright of published works, whereas PJMS and JPMA grant copyright to the authors. JCPSP also publishes case reports and evidence-based reports, a characteristic lacking in the other two journals. Ownership and operational models also vary, with JCPSP being institutionally owned, PJMS functioning on a business model, and JPMA is an official publication of the Pakistan Medical Association. Concerning plagiarism, JCPSP and JPMA use TurnitIn, while PJMS employs iThenticate. PJMS receives the maximum submissions per month and has the highest rejection rate. Impact factors are also different, with PJMS having the highest IF. As per Journal Citation Report released in 2024 by Clarivate the Impact Factor and Quartile ranking of these three journals is as under:^1^

Peer Review processes also differ, with JCPSP and JPMA employing double-blind peer review, in comparison to PJMS’s open peer review system. JCPSP also offers honorariums to reviewers, while PJMS provides publication discounts and honorarium to a selected few reviewers, and JPMA does not offer any incentive to the reviewers.

Commitment, integrity, and teamwork are fundamental to the achievements of JCPSP, JPMA, and PJMS. Among them, PJMS excels in administrative efficiency, its structured incentives and waivers policy, as well as organizing workshops and conferences for the medical community. JCPSP stands out for its flexible author withdrawal policy, comprehensive internal review process, and well-defined authorship guidelines. JPMA’s notable contributions include dedicated student sections, the publication of KAP studies, Narrative Reviews, Experimental Animal research, and Lab Diagnostics. It is highly commendable that both PJMS and JPMA actively support medical journalism by offering training opportunities to students and healthcare professionals.

## Authors Contribution:

**AE** wrote the first draft of the article which SAJ subsequently revised. Both authors approved the final submission.

## References

[1] Meo SA, Jawaid SA (2024). Journal Citation Report 2024 and Impact Factor of Pakistani Biomedical Journals:Grading impact and analysis. Pak J Med Sci.

[2] Farasat N (2024). Internship during Certificate Course in Medical Editing by UHS and PAME. Pak J Med Sci.

[3] CPSP will encourage all such academic activities to promote the discipline of Medical Journalism.

